# Mr. Guthrie's Lectures on the Operative Surgery of the Eye

**Published:** 1823-12-01

**Authors:** 


*Lectures on the Operative Surgery of the Eye.* By J. G. Guthrie, Esq. &c. &c.

[Concluded from page 448.]

Our readers are aware that, in our last number, we concluded the subjects of Entropium and Ectropium so fully treated of in Mr. Guthrie's valuable lectures on the Operative Surgery of the Eye. We now proceed to notice the other topics of the volume, in the order in which they stand.

1. *Cohesion of the Edges of the Eyelids, or of the inner Part of the Lid to the Ball of the Eye.* These two are generally found combined, and the complaint is of a very intractable nature. The structure and vital properties of the conjunctiva, as a sero-mucous membrane, do not admit of adhesion taking place between its surfaces whilst they remain entire, whatever degree of inflammation may be excited on them.

"In consonance with this opinion, we never find that union of these parts occurs in any of the inflammations arising from other than mechanical causes, unless ulceration has previously taken place; a provision which Nature has made for the preservation of vision, which would otherwise be lost in every severe case of inflammation. The causes, then, of these adhesions are generally to be found in the application of such substances as abrade or destroy the conjunctiva covering both surfaces; such as melted lead or other metals, the mineral acids, quick lime, and scalding water; this also occurs from burns, and from wounds affecting both surfaces. Either of the substances enumerated getting between the eye-ball and the lid, certainly abrades the surfaces of both, whilst it generally gives rise to ulceration, and often to sloughing of the cornea, when the termination is usually fatal to the eye. In all these cases the inflammation which takes place is severe, and from the cornea being implicated, the intolerance of light is so great as to prevent the patient, when neglected or improperly treated, from attempting to open the eye; and the abraded or ulcerated surfaces are kept for an indefinite time opposed to each other. At a period which cannot be precisely indicated, means of union are offered by the inner surface of the eyelid; and, if I may use the expression, are accepted by the surface of the eyeball; for I am of opinion that the whole of the matter supplied to effect the union is furnished from the vessels of the lid; which hypothesis seems to be confirmed by an examination of the substance connecting these parts in the slighter and relievable cases, which is always found to partake of the nature of the lid, and perhaps of the conjunctiva of the eyeball, but not of the cornea; and which may account for the fact that I have never met with any difficulty in effecting a permanent separation of such partial adhesions from the cornea, whilst it is next to impossible to do so by any means we are at present acquainted with, when union has taken place between the conjunctiva lining the lid and that covering the sclerotica alone." 90.

When the attachment takes place by one or more narrow slips of communication, an amelioration may frequently be effected by the simple operation of dividing the attachment close to the eye, and cutting it away from the lid; but if it arise from the angle of reflection, it will acquire repetition, great care in the treatment, and still will not be completely successful.

"I have already," says Mr. Guthrie, "expressed my opinion as to the impropriety of interfering with these adhesions, when they are broad, and proceed from the reflection of the conjunctiva from the eyelid to the eyeball; when this union is, however, narrow, and the adhesion has become elongated, so as to assume the form of a band or bands, having distinct formations and attachments, they may be cut away with partial success, but will seldom be completely removed; and this partial success will only be attained after two or more operations, which are to be accomplished in the following manner: the eyelid, if the lower one, and such adhesions are usually formed in it, is to be drawn outwards, so as to give a full view of the membranous band attaching the parts to each other, which is then to be divided close to the cornea, and down to the reflection of the conjunctiva, by one snip of a pair of scissors. The eyelid being now completely drawn outwards, the portion of the membrane which remains attached to the conjunctiva is to be carefully raised with a forceps, and cut away in like manner with a pair of scissors. In order to prevent adhesion, the cut surfaces of the eyelid should be lightly touched with the sulphate of copper, and the patient should frequently roll the eye and move the lid for the first twenty-four hours, during which time it may be washed with the decoctum papaveris albi. The attention must now be directed to the healing of the part next the cornea, and the edge of the lid, whilst it should be prevented as much as possible at the angle between the eyeball and eyelid; in other words, the wound should be induced to heal downwards rather than upwards. There is never any difficulty in clearing the cornea; but it is very much the reverse with the conjunctiva beneath it; for, although the cicatrization may be nearly accomplished, with little appearance of adhesion, still, on its becoming complete, a contraction takes place, which in a great degree restores the appearance of the previous attachment. By a repetition of the operation, some further advantage may be gained; but I believe I may say, the parts are never restored to their natural state, although the deformity and inconvenience may be almost entirely removed." 95.

On the section on "Wounds of the Eyelids," we need not dwell. They are neither dangerous nor difficult of cure. They are to be treated in the same manner as wounds in general. When the lachrymal canal is divided, Mr. Guthrie doubts very much if union ever takes place in such a manner as to render it pervious. Wounds and injuries of the forehead are often productive of deformity, and sometimes of blindness—they consequently demand considerable attention.

"Vision is sometimes affected by these wounds in a manner that would appear unaccountable if we were not acquainted with the subtlety of nervous sympathy. The relation of the numerous cases I have met with of this nature would rather gratify curiosity than answer any useful purpose—from the wind of a cannon ball (as it is said) to the prick of the point of a sword. It appears to me, however, from a consideration of these cases, that defective vision, or a total deprivation of sight, rarely takes place, unless the injury immediately affects a branch of a nerve, of such magnitude as to be usually demonstrated in a common anatomical dissection. These wounds or injuries are either situated, for the most part, on the forehead towards the nose, inplicating the supra-orbital branch, and the nasal branch of the first division of the fifth pair of nerves, or immediately below the eye, affecting the infra-orbital branch of the second division of the same nerve. It is not necessary that any injury should have been inflicted on the eye itself, and the absence of all appearance of it at the moment, independently of the patient's own knowledge on the subject, sufficiently attests the fact, that the amaurotic affection is the result of sympathy, and not of direct injury to the eye. In some instances, the deprivation of sight would appear to be simultaneous with the receipt of the blow; in others it comes on more slowly, during the process of healing, and is seldom complete; whilst in a third set, it only commences after the circatrization is fully accomplished." 100.

When the deprivation of sight is instantaneous, our author suspects, notwithstanding many opinions to the contrary, that the eye itself has suffered from the general concussion. Beer and Weller place great reliance on the appearance of the iris as indicative of the nature of the derangement—that is, whether it has arisen from sympathy with the supra-orbital nerve, or from a direct affection of the eye. They consider a contracted pupil to indicate concussion of the eye—a dilated pupil, a laceration of the supra-orbitary nerve—inferences which do not accord with Mr. Guthrie's experience, inasmuch as the appearance of the eye has been more natural in the amaurosis from sympathy than from direct injury.

When complete blindness takes place as the immediate consequence of a blow on the eye or the forehead, and continues without amendment for several days, it has hitherto, Mr. Guthrie believes, been found incurable. When the defect comes on slowly some days after the receipt of the injury, the case is less desperate, though a cure is equally uncertain. The complaint may be arrested, but is seldom entirely removed. If internal inflammation, acute or chronic, supervene, there is no hope of the affected eye, and great danger of the other also.

"When the injury committed on the forehead is sufficiently severe to affect the brain as well as the supra-orbitary nerve, it may not only give rise to symptoms indicating inflammation of the brain and its consequences, but to a derangement of the nervous system of the eyes which may show itself in an amaurotic affection of both. I consider an amaurosis from injury to be much on a par with cataract from injury, and often occurring without affecting the other eye, whilst both diseases, when caused by internal derangement, seldom exist long alone.

"When the defective vision follows a wound on the forehead, the only hope of relief that we are at present acquainted with lies in a free incision made down to the bone in the direction of the original wound; and even of the efficacy of this I am sorry I cannot offer testimony from my own practice; having failed in every case in which I tried it." 103.

In all cases of wounds of the forehead, accompanied or followed by defect of vision, or amaurosis, we should endeavour to ascertain whether it has been caused through nervous sympathy, injury of the eye, or concussion of the head. It will also be useful to ascertain whether the amaurosis, subsequent to the commencing cicatrization, be a consequence of nervous sympathy alone, or of a low irregular vascular action going on in the internal part of the eye, which is to be recognized by its symptoms and appearances; while the pure nervous derangement is only discoverable by its effect in destroying vision.

"The object of this particular inquiry is to regulate the practice to be followed, and particularly in relation to that recommended by Beer; for every wound of the forehead implicating the fifth pair of nerves does not cause amaurosis; whilst many cases of defective vision, accompanied by wounds, are dependent on other injury than that committed on the forehead.

"According to my observation, the eye, when amaurotic through injury of the supra-orbitary nerve, often shows little or no derangement of structure, the iris preserves more or less of its natural motions, and the information we acquire, is from the patient's declaration of his loss of sight, which is generally unaccompanied by pain. When amaurosis is the consequence of a direct injury to the eye itself, there are, for the most part, positive marks of the injury having been inflicted upon it, which cannot be mistaken; such as extravasations in, or lacerations of some of the internal parts. The motions of the iris are greatly impaired, the pupil is dilated, the pain is considerable. When the amaurosis is the consequence of concussion or of derangement within the head, such as extravasation of blood or deposition of matter, the eye of the side opposed to the injury is most frequently affected, when both are not equally implicated, and which state will be easily recognized by those conversant with injuries of the head, by whom alone they should be treated.

"Wounds of the forehead should be managed according to the principles of surgery applicable to wounds of the head in general. If loss of sight should have immediately followed the injury, an incision should be made down to the bone at the part affected, so as to render it a clean incised wound, whether it were before a contused or a lacerated one, which should then be dressed simply, and suppuration encouraged in a moderate degree, by the application of a poultice. If the amaurosis should only appear after cicatrization has commenced, the same kind of incision should be made on that side of the wound nearest the supra-orbitary foramen. If these should be found of no avail after a few days, the operation of dividing the nerve, just above where it passes out from the orbit, should be attempted, as a last resource; and those who consider that this operation fulfils the intention better than dividing the branches, which are irritated at the part itself, will have recourse to it at once in the manner recommended by Beer, which will certainly be preferable when cicatrization has nearly or completely taken place, and a new wound must in any manner be made. When the injury has been inflicted upon the eye, and the amaurosis is complete, or commences slowly, or after a few hours begins to subside, a division of this branch of the fifth pair of nerves must be useless, and may do mischief. In this case, independently of any considerations which may arise from injury committed within the head, recourse may be had to general bleeding; cupping on the temple of the affected side; a regular drain by the application of four or six leeches near the eye, every day, or second day; purgatives; abstinence; and, if applications are made to the eye, I consider the decoctum papaveris albi, used warm, the best local remedy. At a subsequent period, stimulants, galvanism or electricity, may be useful, if a partial restoration of vision be effected. If signs of active inflammation take place, the depletory means must keep pace with them; but when the low irregular action occurs, to which I have alluded, dependence must be placed on local bleeding, blisters behind the ear, a caustic issue above it, and mercury given in such manner as to affect the mouth quickly." 105.

When amaurosis is followed by inflammation, the matter is much more serious than when the complaint is apparently nervous—and that because the other eye is so likely to be attacked by internal inflammation. Our author has two cases of this kind under his care at the present time. In one, the wife knocked her husband down with a frying pan, rupturing the iris by the blow. In the other case the husband knocked the wife down with a pair of bellows, rupturing both cornea and iris. Low inflammation has, with amaurotic amblyopia, taken place, in both instances, in the originally unaffected eye. The man will probably lose both his eyes—the woman is likely to be more fortunate in having one eye left with sight.

"The pain experienced in affections of this nature is not so much felt in the eye as it is in the different branches of the supra-orbitary nerve, near the nose over the forehead, and round the side and even back of the head, showing that the sympathy is as complete between the eye and the nerve, when the former is suffering from internal inflammation, as between the nerve and the eye, when the affection of the nerve is the cause of amaurosis." 106.

Mr. Guthrie has another case under his care, of a curious and mixed character. A gentleman was accidentally shot in the face by his companion, seven of the shot striking the forehead and side of the head, while one was supposed to have entered the orbit by the side of the eye. Complete blindness was the immediate consequence, from which he gradually recovered in part, but not so as to distinguish a letter in an octavo page. The small holes made by the shot soon healed. In this state he came under Mr. Guthrie's care, the eye being, apparently, as sound as the other, without pain, and attended with no other inconvenience than from the pressure of the hat on the places where the shot had entered. He considered his vision to be more defective at intervals, from a black cloud (as he expressed it) passing over the eye, and descending from the outer corner of it. This cloud, in the course of treatment, diminished, so as not to come over the eye, except after reading. In this case Mr. Guthrie removed the shot that caused uneasiness; drew blood from the temples by cupping, and had recourse to purgatives and stimulants, assisted by alteratives and abstinence. There is every appearance that the cure will be complete.

The next section is on tumours of various descriptions affecting the eye-lids—hordeolum—chalazion, grando, &c.

On hordeolum, or stye, we need not dwell. When the inflammation which gives rise to this affection does not proceed to suppuration, the swelling soon degenerates into a hardened tumour, which has obtained the appellations of chalazion, grando, lythiasis, tophus, &c. It is commonly a small fleshy tumour, which is sometimes very troublesome, occasionally dangerous, and generally requires an operation for its cure.

"The removal of this swelling may be occasionally effected when it is small, by gentle friction with any smooth solid substance, such as an old woman's gold ring, which has long obtained credit for this service, and sometimes by stimulant applications, which, whilst they induce inflammation and suppuration, occasionally bring on unhealthy action, and give rise to sores of a malignant character. When a chalazion becomes troublesome from its size, it should be removed by making an incision through the skin over it, in the direction of the eyelid, of sufficient extent to expose the tumour, which should then be raised with a hook, and cut off with the scissors, care being taken not to injure the cartilage, or the edge of the lid; and if a portion should adhere to either, it must be left, and will be destroyed by the suppuration which will ensue. Escharotics generally do mischief, and ought, therefore, to be avoided, as capable of giving rise to cancerous ulceration in persons of a cachectic habit. Professor Beer seems to consider this as a common occurrence in Germany; but in this country, where the practice of surgery is in general much better understood, and relief is speedily obtained, such a change is very rarely observed. When it does take place, it will, I suspect, be only symptomatic of a cancerous diathesis, which has already shown itself by local derangement in those parts most liable to it, and the appearances of its commencement on the eyelid can neither be mistaken nor cured." 110.

The most simple kind of tumour forms under the skin of the lid, elevates the part, and causes it to look like a little flat wart. An incision is to be made across the centre of the little tumour to its middle—the contents are then to be squeezed out by pressure made at the base by the nails of the two thumbs, and the cure is generally completed in 24 hours. When these tumours are situated under the orbicularis muscle, they may still be removed in the same manner; but if they adhere to the tarsal cartilage, the eyelid should be everted, when the projection of the tumor will be seen through it, into which an incision should be made, and the contents are to be pressed out in the same manner as beforementioned.

This method of removing tumours through an opening in the cartilage is never attended with any inconvenience, and ought always to be adopted when the appearance of the inside of the eyelid is changed from its natural colour to a semitransparent yellowishness, showing the firm attachment of the tumour to the cartilage, even if it do not indicate its partial removal by absorption. The head being firmly held, the eyelid is to be everted and retained in that situation by the fore-finger of the left hand; the surgeon makes an incision a little longer than the projection of the tumour, with a small sharp-pointed knife, the hand being unsupported in order to avoid an accident from the starting of the patient. When the cartilage is divided, the tumour appears, and a touch of the knife discloses its nature, whether fleshy, fluid, or otherwise. If fluid, the contents escape, and the operation is completed by introducing the blunt end of a common probe into the sac, and moving it about in every direction, so as to empty it completely, and at the same time to excite inflammation. This should be repeated every day for three, four, or five days, at the end of which time the sac will have become consolidated, and the opening in the cartilage nearly closed. The little remaining tumour or hardness soon disappears, never to return in the same place from the same cause." 114.

We shall here introduce an extract relative to a different kind of tumour.

"Whilst the last-described tumours affect for the most part the immediate vicinity of the tarsal cartilages of both lids, the atheromatous ones are usually situated at some distance from them, although more generally in the upper lid: they occur equally without pain, but feel rounder, more elastic, and deeper seated; they roll more readily under the finger, and seldom adhere to the skin so as to cause inflammation, unless of long standing, or when they have been irritated. If a small opening be made into one of these tumours, and pressure is applied, the contents are readily made to pass out in the shape of a tape-worm, and resembling the medullary matter of the brain. It is, however, secreted very rapidly, not only by the whole sac, but even by a part of it, which renders the removal of the whole of it necessary to prevent a recurrence of the complaint. If the tumour be small, an incision should be made over it, extending beyond it each way, and the skin separated so as to allow the tumour to rise on pressure. The sac, which is usually rather thick, should now be raised with a hook and carefully dissected out. If the tumour be large, an incision should be made directly into it, so as to allow the contents to escape; or if the operator should have fairly divided it, unconscious of its nature, the two sides of the sac may be laid hold of with the forceps and dissected out, which will be assisted by making pressure below the tumour, so as to press the inside of the sac upwards and outwards: if any portion of it should remain, it ought to be touched with the argentum nitratum, or other caustic, and the wound should not be allowed to close until every appearance of a further secretion from the sac has ceased." 115.

Encanthis.—This consists in an enlargement of the caruncula lachrymalis, implicating the valvula semi-lunaris, extending (when inveterate) along the edge of either eyelid, and advancing as far forward on the conjunctiva as the cornea. It is by no means of frequent occurrence in this country, in any form, and is very rarely seen in a scirrhous condition. It is usually the consequence of neglected or ill-treated inflammation of the caruncula lachrymalis arising from external injury or irritation.

"The treatment of simple inflammation of the caruncula lachrymalis must depend upon the nature of its more obvious cause; and, as it most frequently arises from the presence of an irritating substance, the part itself, as well as the inside of the eyelid, should be carefully examined, and every extraneous substance removed. Dr. Monteath says he has seen it originate twice from a loose eyelash sticking in the superior punctum lachrymale and irritating the caruncle. When no immediate exciting cause can be discovered, several leeches should be applied in the neighbourhood of the part, and one should certainly be placed on the inside of the lower lid, close to the inflamed caruncle, the bleeding from which should be encouraged by repeated fomentations with warm water; and I would advise a repetition of the leeches until it is evident that the progress of the inflammation towards suppuration cannot be prevented, when a light poultice of bread and water should be had recourse to until the abscess is duly formed. I do not coincide in the opinions of foreign writers who recommend cold water and cold applications, generally, instead of the more active practice I have advised; and which may perhaps be one cause of the great frequency of the disease abroad, compared with its occurrence in England. When suppuration has unfortunately taken place, the treatment should still be, in the first instance, of the mildest nature, followed up by slight astringents; but if these should not be found sufficient to prevent the appearance of the fringed-like or fungous growth alluded to, recourse must be had to the infusum sabinæ, the sulphas cupri, and the argentum nitratum. The Germans recommend that they should be sprinkled with a powder composed of sacchar. alb. ʒij. aluminis usti gr. xv. zinci sulph. gr. iv. but in this, I place little comparative confidence." 121.

Pterygium. The symptoms and appearances of this complaint are so well known, and have been so often described by surgical and ophthalmic writers, that we need not dwell on them here. The cure of the well-formed pterygium should always be accomplished by removal. The pterygium tenue may be sometimes cured, when the patient is afraid of an operation, by slight scarifications and stimulants. The scarifications ought to be made by raising the pterygium with the forceps, and cutting across it in two or three places. After the second day the solutio argenti nitratis, or the vinum opii, or a solution of the carbonate of potash in distilled water (20 grains to the ounce) may be resorted to; but the cure by these means occupies a great deal of time—is often incomplete—and the patient generally is obliged to submit to the operation after all. The cure by this last is perfect, as far as relates to the termination of the disease; but a slight defect remains on that part of the cornea where the apex of the pterygium was situated. This arises from the cicatrix which takes place where the conjunctiva was removed, and which is in fact an indelible mark or nebula, although much less than the extent of surface exposed by the operation. The following is the mode of operating preferred by our author.

"The patient, being seated lower than the operator, ought to rest his head against the breast of an assistant, who elevates the upper eyelid, and fixes the eye with the fore and middle fingers of one hand, whilst he depresses the lower eyelid with the other. The surgeon should then desire the patient to turn the eye outwards, if the pterygium arise from the inner canthus; and, whilst it is thus on the stretch, take the opportunity of grasping it between the points of the forceps, about two lines from the cornea, and then raise it from the sclerotica, until he has room to pass an iris, or spear-pointed cataract knife, underneath it and to the inside of the forceps, when it is to be cut through from within outwards. The extremity of the pterygium being still held by the forceps, will allow the operator to cut it off close to the cornea, with the same knife, or by the curved scissors, which will be more easily effected, and more completely done. If any portion of the edge of the pterygium should have been left after this operation, it must be removed by the scissors, or it will keep up for several days the subsequent suppuration, and the eye should be washed with warm water until the bleeding ceases, when a compress and bandage should be applied, so as to keep the eyelids closed, but not to press on the eye. The eyelids should be carefully washed clean night and morning, or oftener if it be found agreeable to do so; but the eye should be opened to examine the wound, only so far as will show that the conjunctiva is not highly inflamed, or that chemosis has not taken place. On the fifth day the eye should be examined, when the divided surface of the sclerotica will be found covered with a yellow-coloured deposit resembling mucus, whilst the surrounding edge of the conjunctiva is irregular, red, and inflamed. This gradually diminishes, the wound contracts, and a new membrane is formed on the part, which, after a time, becomes less opaque, and assumes more and more the natural appearance of the conjunctiva. This process of the cicatrization, which goes on from two to three weeks, requires no assistance from art, and should not be interfered with by any application, unless it appears that some irregularity or elevation is about to take place, when this spot should be lightly touched with the sulphate of copper or the argentum nitratum. When the fungous elevation has been small and well defined, I have removed it with advantage by a snip of the scissors. When the cicatrization is nearly completed, mild stimulants should be dropped into the eye, to remove the chronic inflammation which may remain, and to assist in the absorption of the base of the pterygium, which is almost entirely effected, so as in time to be scarcely, if at all observable." 132.

The true pterygium rarely assumes a malignant character. When it does, the termination of its sides in the surrounding conjunctiva will not be so well defined—red vessels will be observed passing from a distance into it—its colour will have changed to a darker red—it will be found to adhere firmly to the cornea and sclerotica beneath, so as not to be raised from them—it bleeds on being slightly touched—and is always attended by pain, sometimes of a lancinating nature, shooting through the eye, and indicating the presence of a cancerous disease, which can only be cut short by the removal of this part of the eye.

Removal of extraneous Substances from the Eye. The entrance of any extraneous substance is quickly perceived, not only by direct sensation, but in consequence of the motion of the lids. The situation of the offending body, however, has great influence in modifying the degree of pain produced by its presence. Thus, if the foreign substance should be lodged in that part where the conjunctiva is reflected over the ball of the eye, especially in the upper lid; then the irritation which ensues is merely that which arises from the local injury inflicted on the particular portion of the conjunctiva affected by it; whereas, when a solid substance sticks in the cornea, or the anterior and most prominent part of the sclerotica, over which the tarsal cartilage passes in winking, more severe symptoms ensue. If the foreign body should stick in the sclerotica at a part which is not pressed upon by the tarsal cartilage in winking, the symptoms are frequently so slight as only to cause an inconvenience scarcely amounting to pain.

"I lately removed," says Mr. G. "a small piece of the husk of a grass seed from the sclerotica at the upper edge of the cornea, in the eye of a young medical student, which had been lodged there for sixteen months. It had merely given rise to a little occasional irritation, which was augmented on his taking cold; and three or four red vessels running from the spot were then increased in size. It had caused a small depression, in which it was lodged, but whence it was easily removed, and all inconvenience immediately subsided. A knowledge of the comparative impunity with which such substances may be retained in the folds of the conjunctiva, especially of the upper lid, will cause a closer investigation to be made of this membrane, when the origin of the chronic inflammation, for which relief is desired, is attributed to an injury or accident of this nature; and if any fungous excrescence is perceived, it should be removed to its base by the scissors, in order that no extraneous matter may be concealed within it; for the records of surgery afford numerous examples of this fact, and several cases have occurred to and have been happily cured by myself; the cure, indeed, after the removal of the exciting cause, being accomplished in almost every instance by the unassisted efforts of nature.

"Whenever the admission of any irritating matters between the eyelids is suspected, they should be carefully examined; the lower one offers no difficulty, the upper lid should be everted in the following manner. The patient should be placed on a chair, lower than the surgeon; who, taking hold of four or five of the cilia in the centre and close to the edge of the eyelid, gently draws them downwards and outwards from the ball of the eye. A probe, or other small round or blunt instrument, is then to be placed horizontally across the lid, immediately above the upper edge of the tarsal cartilage, when, by raising the cilia, which have been already grasped by the fore-finger and thumb, the eyelid may be turned out over the probe; the offending matters will be in general found adhering to the everted surface. If the patient be desired to look downwards with the other eye, the whole of the conjunctiva may be readily examined.*

"When a small fly, or any substance which is not sufficiently pointed to stick into or fast to the conjunctiva, gets between the lids, there is an action set up in the orbicularis muscle for its removal, which, together with the flow of tears, will frequently be successful, provided the person will remain perfectly quiet, and not strain the eye; at the end of a few minutes it will be found at the edge of the lid, and may be readily removed by a bystander." 137.

There never can be much difficulty in discovering foreign bodies that penetrate the cornea—and they must, of course, be removed. Wounds of the cornea made by a sharp cutting instrument, and of small extent, unite without difficulty, and leave no mark. If of large dimensions, it heals with a little elevation or irregularity, and a trifling scar remains. We must pass over many pages of judicious observations on wounds, &c. of the eye, and also a whole section on "tumours which are formed within the orbit, and on protrusions of the eyeball," because these portions cannot be concisely analyzed, and extracts would not do justice to the work. We come therefore to a chapter "on the alterations of form in the anterior part of the eye.—*Staphyloma.*"

---

* "When surgical aid is not at hand, I have seen ladies in the country slightly raise the eyelid in the same way, and introduce the tip of the tongue underneath it, by which a foreign body may be often brought out. The sensation is peculiar, but not unpleasant."

This disease is known to take its name from its supposed resemblance to a grape, and consists essentially in a protuberance of the anterior part of the eye-ball, particularly of the cornea, which has become opaque in consequence of acute inflammation, assuming various appearances all bearing a general resemblance to each other.

"When the cornea does not project between the eyelids so as to prevent their closing, it causes but little inconvenience to the patient, and is only unpleasant from the deformity it occasions. The first and most simple kind of staphyloma is of this nature, and appears to have been overlooked by both Richter and Beer in the definitions they have given of the disease. It occurs after or during a long-continued inflammation of the eye, which has principally affected the conjunctiva and cornea, during which the latter becomes opaque, loses its natural appearance, projects, and becomes more spheroidal, the conjunctiva covering it is thickened, and red vessels are to be observed on its surface, the iris may be partly discerned in its natural situation through the semi-opaque staphyloma; the anterior chamber contains a larger quantity than usual of the aqueous humour, and the projecting cornea has the greatest resemblance to an equal portion of a white grape. This state of the eye is irremediable.

"There is a good illustration of this disease in the person of William Jackson, now attending at the Infirmary. When he first presented himself for advice, the eyelids were enormously thickened in consequence of a granulated and fungous state of the conjunctiva; the eyeball appeared to be a red fungous mass, in which the cornea was not distinguishable: he was perfectly blind. In the course of twelve months' treatment, the eyelids have been reduced to their natural state, the conjunctiva covering the eyeball has a more healthy appearance, the cornea is now well defined, semi-transparent like horn, and the patient can see, so as to avoid large objects in his way, with the left eye. The cornea of the eye projects in a spheroidal form, and the anterior chamber is increased in size. The disorganization having proceeded further in the right eye, renders it entirely irrecoverable." 168.

By the German writers, staphyloma is supposed to depend on an external and internal swelling of the cornea, the consequence of inflammation, accompanied by a corresponding swelling and projection forwards of the iris, which adheres to the cornea, constituting the first step in its formation. For its completion they suppose that the secretion of the aqueous humour in the posterior chamber must continue unimpaired, when the cornea yields to the pressure, enlarges, and becomes staphylomatous.

"The thickness of the cornea should rather be regarded as a means offered by nature for resisting disease, than of assisting in its formation, which appears to me to be proved by the fact, that the remark of the greater prevalence of staphyloma in children, has not been correct for the last twenty years, since the general introduction of vaccination, whilst it must be admitted with great regret by every surgeon of every hospital or public charity in the kingdom, that, during the last two years, infants have suffered considerably from it in consequence of the greater prevalence of the small pox. The reason why the inflammation of small pox causes staphyloma more frequently than any other inflammation, is not that there is any thing specific in it, but that it goes on to ulceration, and a state of ulceration of the cornea is necessary in almost every instance for the production of that kind of staphyloma of which we are now treating, and which Richter and Beer suppose to depend on the mere swelling, approximation, and adhesion of the cornea and iris. The anterior surface of the iris and the internal membrane of the cornea are not prone to adhere to each other, they are almost incapable of effecting it by the adhesive inflammation, and, in order to accomplish it, the inflammation must proceed beyond that point. So long then as ulceration does not take place and penetrate the cornea, will an infant, in almost every case of violent purulent ophthalmia, be free from danger of staphyloma; and as often as a variolous pustule forms on the cornea will an infant, in consequence of its ulceration and sloughing, be in danger of staphyloma. The thickness of a staphyloma which has been formed in infancy is readily accounted for, and so is the general adherence of the iris to it. For the opposite reason, a staphyloma formed at adult age is generally thinner and harder; whilst in children it is thicker, more spongy, and softer, but becoming occasionally with time, hard, and almost cartilaginous." 170.

The purulent ophthalmia which formerly prevailed to a considerable extent in a part of the army, gave rise to many total staphylomatous formations; but Mr. Guthrie has reason to believe that this did not happen in a single instance, unless sloughing or ulceration of the cornea had previously taken place.

The cure which Nature endeavours to effect in this disease is by thinning one particular part until at last it bursts, and the aqueous humour is evacuated. But this bursting has been produced by progressive absorption, which ends in ulceration that remains for several days, by which a drain is established, inflammation is excited, and an alteration takes place in the actions of the secretory apparatus, whereby the secretion is diminished. The eye becomes smaller, the ulcer heals—or, if the ulcer and inflammation be more extensive, suppuration takes place internally, and the eye sinks. But we must pass over many important practical observations of our author, as we have only room for one more extract relative to the operation for staphyloma.

"The operation is to be performed in the following manner: the patient's head being secured and the upper lid properly raised, either by Pellier's speculum or the finger, the eye should be steadied by a gentle pressure, or, in a conical staphyloma, by a hook, whilst the operator passes a broad spear-pointed cataract knife across the centre of the staphyloma, and carries it downwards or upwards until rather more than one half of it is cut through by the passing out of the knife. Without allowing the eyelid to fall, the operator should raise the flap with the forceps, or desire it to be done by an assistant, if he should prefer elevating the lid himself; and then cut through the portion of the cornea remaining to complete the circle, with the scissors; when the operation will be finished, and the lid may be allowed to drop. The patient should be desired, if an adult, to be as quiet and as steady as possible; for the slightest irregular pressure or exertion, will cause the escape of the lens, and perhaps of a portion of the vitreous humour, which indeed, in children, can seldom be avoided.

"If the constant irritation should have caused a varicose state of the anterior part of the eye, as shown by the bluish leaden appearance of the sclerotica, which seems to be penetrated close to the cornea by many tortuous dark red vessels, accompanied, in its more advanced state, by a bulging out of particular parts in this same situation, the anterior portion of the eye ought to be removed, and with it the vessels which are in a varicose state. This operation is to be done in a similar manner, although further back towards the centre of the eye, when the whole of its contents will be evacuated. In these cases a weeping venous hæmorrhage frequently takes place, and will continue for hours: it is seldom or never dangerous, unless the opening into the eye has been small, and the choroid coat and the retina, together with any coagulum which may form, close it up, when the bleeding continuing internally, and being only partly discharged, causes pressure and gives great pain. This will be remedied by removing the coagula, by cutting away any portion of the membranes which may fill up the opening, and by the application of cold. I have seldom applied any dressing but a light compress and bandage; if much tumefaction appeared in the lids, attended by pain, I have occasionally had recourse to a poultice. The upper eyelid should not be raised for five or six days, as it causes irritation, and the eye does not require examination whilst it can be cleansed without it. As the process of cicatrization proceeds, a small fungous-like granulation will sometimes appear in the centre, and require to be lightly touched by the argentum nitratum." 178.

We had hopes of being able to embrace the subject of cataract in this paper; but our limits, and the number of other articles requiring insertion, prevent us. We shall probably be able to bring the whole analysis to a close in our next.

